# Soft Surface Nanostructure with Semi-Free Polyionic Components for Sustainable Antimicrobial Plastic

**DOI:** 10.3390/ijms222212315

**Published:** 2021-11-15

**Authors:** Shook Pui Chan, Diane S. W. Lim, Arunmozhiarasi Armugam, Guangshun Yi, Yugen Zhang

**Affiliations:** Institute of Bioengineering and Bioimaging, 31 Biopolis Way, The Nanos #07-01, Singapore 138669, Singapore; chansp@ibb.a-star.edu.sg (S.P.C.); dlim@ibb.a-star.edu.sg (D.S.W.L.); armugama@ibb.a-star.edu.sg (A.A.); gsyi@ibb.a-star.edu.sg (G.Y.)

**Keywords:** antimicrobial plastic, sustainable material, surface phase seperation, surface nanostructure, semi-free polyionic

## Abstract

Surface antimicrobial materials are of interest as they can combat the critical threat of microbial contamination without contributing to issues of environmental contamination and the development drug resistance. Most nanostructured surfaces are prepared by post fabrication modifications and actively release antimicrobial agents. These properties limit the potential applications of nanostructured materials on flexible surfaces. Here, we report on an easily synthesized plastic material with inherent antimicrobial activity, demonstrating excellent microbicidal properties against common bacteria and fungus. The plastic material did not release antimicrobial components as they were anchored to the polymer chains via strong covalent bonds. Time-kill kinetics studies have shown that bactericidal effects take place when bacteria come into contact with a material for a prolonged period, resulting in the deformation and rupture of bacteria cells. A scanning probe microscopy analysis revealed soft nanostructures on the submicron scale, for which the formation is thought to occur via surface phase separation. These soft nanostructures allow for polyionic antimicrobial components to be present on the surface, where they freely interact with and kill microbes. Overall, the new green and sustainable plastic is easily synthesized and demonstrates inherent and long-lasting activity without toxic chemical leaching.

## 1. Introduction

**Scheme 1:**
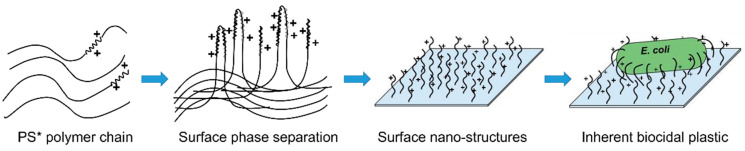
Phase separation occurs on polystyrene surface modified with positively charged (+) antimicrobial component to form submicron scale nanostructures which exhibit inherent antibacterial property.

Microbial infection remains one of the most serious complications and has received much attention in recent years [1,2]. Contamination by microorganisms is of great concern for numerous applications, including in healthcare products [3], medical devices [4,5], food packaging [6,7], water purification systems [8] and manufacturing industries [9,10]. Polystyrene (PS), for example, is a commodity plastics that is widely used for packaging materials or common utilities, however, the contamination on PS surfaces is yet to be resolved. Considerable effort has been devoted to developing self-disinfection polystyrene to avoid using a large amount of disinfectants and for enhanced sustainability [11]. Most strategies focus on formulating PS with other materials, such as chitosan [12] and inorganic materials [13,14,15,16]. Considering the immense scale of PS applications in packaging, especially in the food packaging industry, inherently antimicrobial polystyrene that has a self-disinfecting surface without the drawback of leaching toxic chemicals is highly desired [17,18]. Phase separation in polymer blends or block co-polymers is a widely studied process, and has been used in lithography for various surface modifications [19]. The phase separation of polymer blends has also been used to create an antimicrobial nanostructure by casting-replication processes [20]. Herein, we describe a new method to fabricate submicron soft nanostructures on a polystyrene film surface through a bottom-up approach via the copolymerization of 1% of polyionic components into hydrophobic polystyrene chains. The hydrophilic components self-assemble into different soft sub-micron structures in different solvent systems. The surface nanostructures allow polyionic components, situated at the tip of nanostructure, to achieve greater flexibility and the freedom to interact with the surrounding microbes, thereby granting inherent antimicrobial properties to the polystyrene surface. The resultant antimicrobial plastic (PS*) attracts and kills bacteria via the semi-free polyionic components, functioning in a similar manner to *Drosera* plants, which capture and digest insects upon contact with the plant’s hairy leaf surfaces (Scheme 1).

## 2. Results and discussions

### 2.1. Synthesis and Characterization of Polystyrene Modified with Polyimidazoliums on Surface

**Figure 1:**
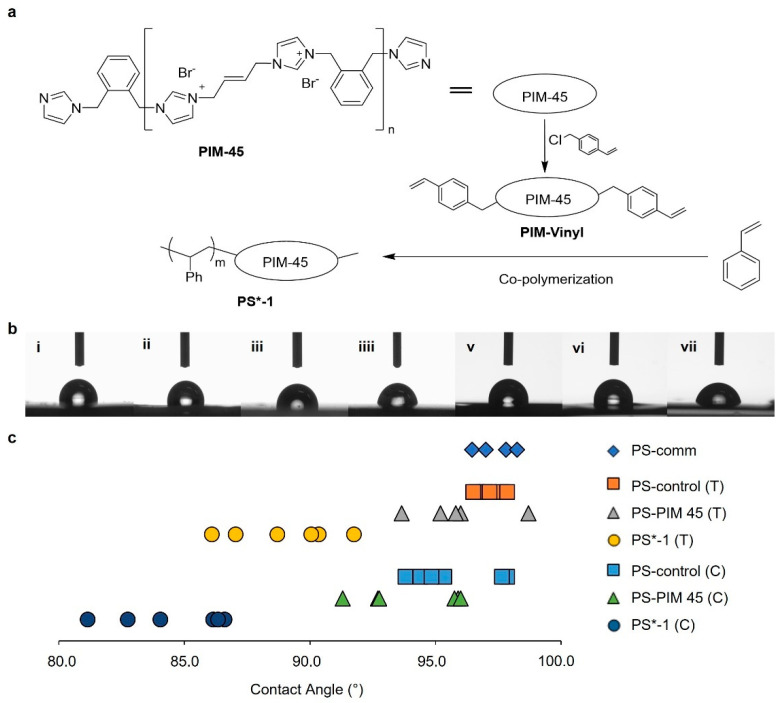
Synthesis and surface wettability of PS*-1. (**a**) Synthetic scheme for the modified polyimidazolium material (PIM-Vinyl) and functionalized polystyrene (PS*-1 with 1 wt.% of PIM-Vinyl). (**b**) Static water contact angle of (i) commercially available PS (PS-comm), (ii) PS-control (toluene), (iii) PS-PIM 45 (toluene), (iv) PS*-1 (toluene), (v) PS-control (chloroform), (vi) PS-PIM 45 (chloroform), (vii) PS*-1 (chloroform). (**c**) Average contact angle distribution of different PS film samples.

To induce phase separation, a polyimidazolium (PIM) compound (PIM-45, Mn~2500 kDa) [21] was selected as the active component to be incorporated into the polystyrene chain. PIM-45 is a main-chain antimicrobial polymer with high selectivity and broad spectrum activity [22,23]. It was modified with styrenyl functional groups at both ends for further copolymerization (Figure 1 and Figure S1). The synthetic approach is illustrated in Figure 1a, where the resultant polymer PIM-Vinyl is subsequently polymerized with styrene and integrated within the polystyrene chain. A series of functional polystyrene film samples (PS*-X, X = wt.% of PIM-Vinyl in polymer) were synthesized via the copolymerization of styrene with different loadings of PIM-Vinyl. PS-PIM 45 was also synthesized by simply mixing styrene with PIM-45 (with no styrenyl end group) as a control. The free PIM-45 can be entirely washed away during the sample preparation process. Signals corresponding to the PIM-45 component within the synthesized polystyrene material can be observed from the ^1^H and ^13^C NMR spectra of PS*-1 (Figure S2). The average molecular weight of PS*-1 is around 49.9 kDa, which is slightly lower than the PS control of 52.2 kDa (Table S1). 

To determine the potential phase separation of PIM incorporated polymers, all PS films were prepared in two solvents, toluene (T) and chloroform (C), with different polarities. Firstly, the surface wettability of synthesized PS was determined by water contact angle measurement. The contact angles of the PS*-1 films were significantly lower than PS controls (Figure 1b,c; Table S2). The PIM compound has high polarity with positively charged imidazolium rings within its structure. A reduction in the contact angle of PS*-1 suggested that the hydrophilic imidazolium group could be mostly presented on the material surface, resulting in a small contact angle.

### 2.2. Surface Nanostructures of PS-Based Materials

**Figure 2:**
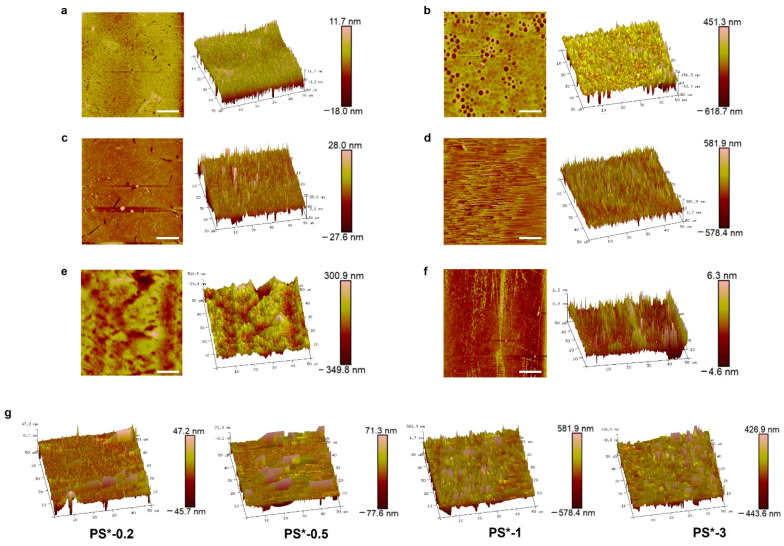
AFM topography images of PS films: (**a**) PS-control (C, chloroform); (**b**) PS*-1 (C); (**c**) PS-control (T, toluene); (**d**) PS*-1 (T); (**e**) PS#-1 (T); (**f**) PS-BZK C12 (T); (**g**) AFM images of PS* samples with different PIM-Vinyl loading amounts (PS*-0.2 (0.2 wt.%), PS*-0.5 (0.5 wt.%), PS*-1 (1 wt.%) and PS*-3 (3 wt.%)). Surface nanostructure was clearly increased from PS*-0.2 to PS*-3. Rough surfaces with different structure arrangement can be observed on PS*-1/PS#-1 samples. Scale bars, 10 µm on AFM images.

The atomic force microscopy (AFM) images exhibit significantly different surface structures between PS-control and PS* samples, Figure 2a–d [24,25]. All PS-control sample surfaces are generally smooth with an average roughness (Ra) in the range of 1 nm < Ra < 20 nm, for which there is no significant difference between both control samples prepared in different solvents [26]. However, PS*-1 samples revealed very interesting submicron scale surface topographic structures. AFM topography images showed that the overall average surface roughness of PS*-1 prepared with chloroform is Ra = 55.1 nm and with toluene it is Ra = 102.9 nm (Table S3). AFM height images showed that PS*-1 sample prepared in toluene has an aligned hair structure, with the height of hairs of around 500–600 nm. In contrast, a PS*-1 sample prepared in chloroform exhibits a honeycomb-like surface structure with a drop from peak to valley of around 800 nm. It is proposed that the surface nanostructures of PS*-1 in toluene or chloroform are PS-PIM co-polymer induced phase separation. The PIM-Vinyl loading in the PS*-1 sample is 1 wt.%, where the number average molecular weight (Mn) of PIM-45 and PS*-1 are about 2.5 kDa and 50 kDa, respectively. It is believed that around 20% of the polymer chains were decorated with a PIM-45 block and that the highly hydrophilic PIM-45 block successfully induced phase separation on the surface to create submicron scale structures. To further verify the relationship between surface nanostructures and the PIM component in functionalized polystyrene, PS* with different PIM-Vinyl loadings were investigated. The surface roughness and the height of the hair structure of PS* increased as the PIM-Vinyl loading increased from 0.2 to 1 wt.% (Figure 2g, Table S4). When the PIM-Vinyl loading further increased, the PS* surface roughness reached a plateau. The density of the nanostructures (amount of hair structures) on the surface were found to be well correlated with the PIM-Vinyl loading. In addition, the peak or bright areas in the AFM height images are correlated to the dark areas reflected in the phase images, which indicates that these areas are relatively more hydrophilic (Figure S3) [27]. These results clearly indicate that the PIM components in the copolymer can spontaneously assemble on the surface, induce phase separation, and accumulates the surface nanostructures. 

Given these findings, this method of inducing nanostructures on PS surfaces was validated using other antimicrobial compounds, a main-chain DABCO-imidazolium copolymer (Mn~2.2 kDa) [28] and small molecules of benzalkonium chloride (BZK), by introducing styrenyl functional moiety to the compound for subsequent polymerization with styrene. Functionalized plastic PS#-1 (with DABCO-imidazolium copolymer) and PS-BZK with a 1 wt.% compound loading were fabricated respectively (Scheme S1). As expected, PS#-1 exhibited similar surface nanostructures and average roughness (Ra = 64.9 nm) to PS*-1, while PS-BZK showed a very smooth surface (Ra = 6.7 nm) (Figure 2e,f, Table S3). This result further suggests that the submicron scale soft nanostructures are induced by the surface phase separation of polystyrene chains, which are decorated with hydrophilic chain blocks. In contrast, polystyrene chains that are decorated with a small molecular compound will not experience a similar surface phase separation, leading to the absence of nanostructures on the material’s surface.

### 2.3. Antimicrobial Property Evaluation

**Figure 3:**
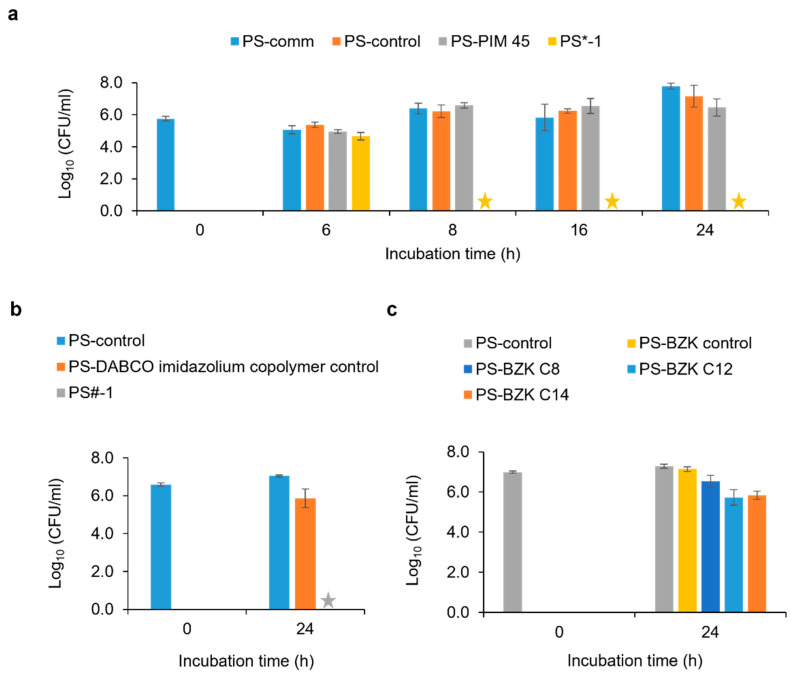
Antimicrobial assessment of functionalized PS with active components based on JIS Z 2801/ISO 22196 test method. (**a**) Time-kill kinetics of PS*-1 against *E. coli* with different incubation time; Antimicrobial activities of (**b**) DABCO-imidazolium functionalized PS (PS#-1) and (**c**) PS-BZK C8, C12, and C14 against *E. coli*. The data are expressed as mean and standard deviations of triplicates. (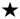) indicates no colony observed on respective samples.

Functionalized PS films (PS*-1, PS#-1) displaying submicron soft nanostructures on their surfaces were then evaluated for their respective antimicrobial properties. All active compounds, before and after the addition of styrenyl functional groups, were first evaluated and proven to have good microbial inhibitory activity, i.e., minimum inhibitory concentrations (MICs) against *E. coli* and *S. aureus* of 4 µg/mL and 2 µg/mL respectively (Tables S5 and S6). The functionalized PS plastic films were subsequently evaluated according to the Japanese Industrial Standard JIS Z 2801/ISO 22196 test method. As shown in Table 1, PS-PIM 45 did not exhibit antimicrobial activity. In contrast, both PS*-1 (T) and PS*-1 (C) exhibited excellent bactericidal properties against *E. coli* and *S. aureus* with a log reduction in the colony forming units of more than 6 after 24 h incubation. The good bactericidal activity indicates that PS*-1 can effectively kill bacteria even when the antimicrobial PIM-Vinyl compound was integrated within the polymer chains. As for fungus *C. albicans*, PS*-1 did not exhibit antifungal activity, but strong fungicidal activity was demonstrated for the PS*-3 surface where the loading of PIM-Vinyl was increased to 3 wt.% (Figure S4). The time-kill kinetics of PS*-1 against *E. coli* with different incubation periods were compared (Figure 3a). PS*-1 exhibited a strong bactericidal property against *E. coli* when the incubation time was more than 8 h. Similarly, PS#-1 exhibited a good bactericidal property against *E. coli* (Figure 3b), while PS-BZK did not exhibit antibacterial activity against *E. coli* for a log reduction <2 (Figure 3c). This result is highly aligned with the AFM observations, whereby PS#-1/PS*-1 exhibited similar surface structures and roughness, while PS-BZK showed a very smooth surface (Figure 2e,f, Table S3). Therefore, we propose that the formation of soft and flexible surface nanostructures in PS films, prepared using co-polymerizing styrene, with main-chain antimicrobial compounds is responsible for the high antibacterial activity observed. In contrast to rigid nano-structured surfaces, most of them do not exhibit a good bacteria killing efficacy using the JIS Z 2801 method, as they are only able to kill the attached bacterial cells [29,30].

To determine the microbial killing mechanism of copolymer PS*-1, a leaching assay was used to assess the leaching properties of PS*-1 following the GB 15979-2002 method with minor modifications made (Figure S5). The results are shown in Figure S6a. The leached solution did not present any bactericidal activity against *E. coli*, which indicates the absence of antimicrobial compounds in solution. In contrast, a bacterial reduction of more than 80% was observed (Figure S6b) when PS*1 was introduced to the bacterial solution. This suggested that PIM-Vinyl was well incorporated in PS*-1 rather than simply embedded within the PS film, thus it did not leach out from the material to cause antibacterial activity. PS*-1, with covalently bonded PIM-Vinyl, inhibited bacterial growth while in contact with bacteria in the solution. In addition, PS-PIM 45 did not reveal any bactericidal activity, indicating that the compound was not entrapped within the polymer matrix. As the PIM-45 compound with no reactive end groups was washed off during the repeated precipitation steps, the resultant PS-PIM 45 material did not contain any remaining antimicrobial materials that may leach out to the test solution to cause bactericidal activity. It should be noted that the length or height of the PS* surface nanostructures (hairs or peaks) are above 500 nm, which provides great flexibility and freedom for the polyionic components in the nanostructures to act as free antimicrobial compounds to kill bacteria [21,22,23].

**Figure 4:**
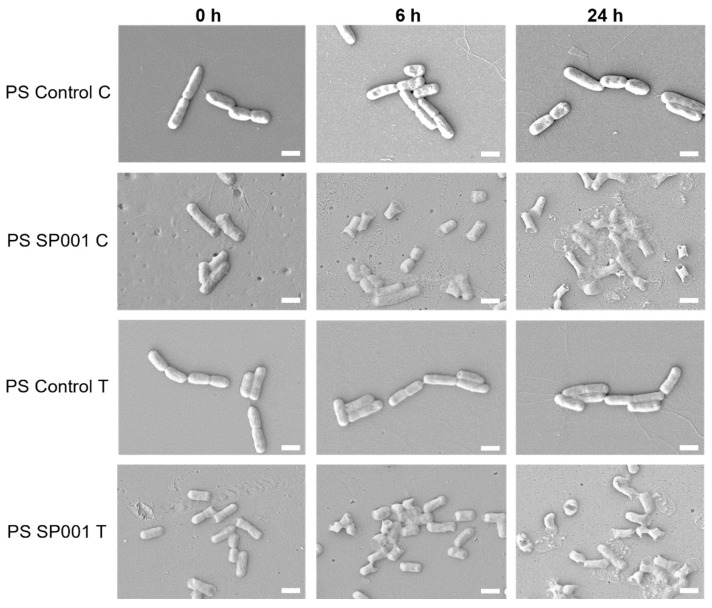
Bacteria morphology changes on PS*-1 surfaces. Cell morphology and viability of *E. coli* on PS-control and PS*-1 surfaces after 0 h, 6 h, and 24 h incubation determined using SEM. Scale bars, 1 µm.

The morphological changes of bacteria on PS-control and PS*-1 surfaces were examined under a scanning electron microscope (SEM) (Figure 4). No changes to the morphology of *E. coli* in the control samples were observed at varied time points. However, a small number of deformed bacteria cells were observed on both PS*-1 films prepared from chloroform and toluene after 6 h exposure to PS*-1 surfaces, where the rupture of bacteria can be observed on the end of *E. coli* cells. After 24 h, all bacteria cells on PS*-1 surfaces were deformed and ruptured, leading to cell death. This further confirmed that the killing of bacteria occurred due to the contact of bacteria with polystyrene surfaces that contained an imidazolium-based antimicrobial compound, which eventually caused membrane lysis [21,22,23].

Therefore, it is suggested that the presentation of polyionic components on PS*-1/PS#-1 surfaces creates soft nanostructures (submicron scale) that can interact with bacteria cells via electrostatic and other forces. The contact of bacteria cells with semi-free polyionic components resulted in the high antibacterial performance of functionalized PS, as demonstrated in Scheme 1. The soft nanostructures and the positively charged antimicrobial component on the surface function as micro-*Drosera* plants that capture and digest insects using stalk hair and the sticky liquid covering their leaf surfaces. 

### 2.4. Durability and Biocompatibility of PS-Based Antimicrobial Plastics

The inherent antimicrobial property or the shelf life of the disinfection effect of PS*-1 was evaluated using an accelerated aging test according to the ASTM F1980-16 test method [31]. PS-control and PS*-1 (T) were stored in a closed environment with a constant humidity of >95% at 60 °C. After two months of aging at 60 °C which approximates to two years in real-time, PS*-1 exhibited excellent bactericidal activity against *E. coli* (Figure S7a). None of the aged PS samples showed obvious physical changes. This suggests that the PS*-1 samples may have an average shelf life of more than two years for different applications.

As for safety concerns regarding PS* materials, a cell viability assay was conducted using mouse fibroblast L929 cells. There is no significant viability difference between PS*-1, PS-PIM 45, and PS-control, with cell viability close to 100% (Figure S7b). This confirmed that PS*-1 did not have a cytotoxicity effect on mouse fibroblast cells. Separately, a hemolysis assay was performed using red blood cells. No significant hemolysis activity was observed on PS*-1 as the average hemolysis percentage at both 5 mg and 10 mg were comparable to the negative control (Table S7). Hence, it can be concluded that PS*-1 is biocompatible, as illustrated in both the hemo- and cyto-compatibility assessments.

## 3. Materials and Methods

### 3.1. General Information

All of the anhydrous solvents and chemicals were used as received from the commercial suppliers, unless otherwise indicated. Styrene (≥99%) (Lot: STBJ7184) and benzoyl peroxide (75%) (Lot: MKCG5941) were purchased from Aldrich (Poznan, Poland). ^1^H and ^13^C NMR spectra were recorded on Bruker (Billerica, MA, USA), AV-400 instrument (400 MHz). Chemical shifts (δ) were reported in parts per million (ppm). SEM images were obtained on a JEOL JSM-7400F scanning electron microscope (Akishima, Tokyo, Japan). AFM topographic images were obtained using a Nanoscope 9.7 Dimension ICON atomic force microscope (Bruker, Billerica, MA, USA) and the results were analysed using NanoScope Analysis 2.0 software (Bruker, Camarillo, CA, USA).

For antimicrobial assessments, tryptic soy broth (TSB) and Mueller Hinton broth (MHB) were purchased from Oxoid Ltd. (Basingstoke, UK), while yeast mold broth (YMB) was purchased from BD (Singapore). The broth solutions were prepared according to the manufacturer’s instructions. Gram-negative bacteria *Escherichia coli* (ATCC 8739), gram-positive *Staphylococcus aureus* (ATCC 6538), Gram-negative *Pseudomonas aeruginosa* (ATCC 9027), and fungi *Candida albicans* (ATCC 10231) were purchased from ATCC (Manassas, VA, USA) and re-cultured according to the suggested protocols. 

### 3.2. Compounds and Materials Synthesis 

The polyimidazolium polymer material, PIM-45 was synthesized using a previously reported method [21,23]. We added 4-vinylbenzyl chloride (3.0 eq) to a solution of PIM-45 (250 mg, 0.122 mmol) in EtOH. The mixture was stirred and heated at 65 °C for 16 h in a sealed vial. After the reaction, the compound was purified via repeated precipitation with THF and dried under reduced pressure at 90 °C. An off-white powder of PIM-45 with vinylbenzyl terminal groups (PIM-vinyl) was obtained. The formation of an antimicrobial compound PIM-vinyl was confirmed by proton nuclear magnetic resonance (^1^H NMR) spectrum (see supporting information).

The synthesis of antimicrobial polystyrene was conducted in dimethyl sulfoxide (DMSO) using dibenzoyl peroxide (Luperox^®^ A75, Arkema, Colombes, France) as an initiator for the polymerization reaction. Dibenzoyl peroxide was purified by dissolving in acetone, followed by precipitation with water before use. In summary, the antimicrobial compound PIM-Vinyl (at the indicated wt.% to styrene) was first dissolved in DMSO prior to the addition of styrene and dibenzoyl peroxide (0.007 eq). The mixture was heated at 120 °C under an N_2_ atmosphere with constant stirring for 16 h. The resulting polymers were purified by repeated precipitation with MeOH and subsequently re-dissolved in toluene. The polymer solution was prepared at different volumes and dried at 90 °C, over 24 h for subsequent testing.

### 3.3. Water Contact Angle Measurement 

The static water contact angle of the PS samples was measured using an OCA15 contact angle analyser (Future Digital Scientific Corp., Westbury, NY, USA). Furthermore, 2 μL of deionized water was dropped on the PS sample surface at a rate of 3 μL/s. The images were captured immediately, and the contact angle was determined. Each sample was analysed in duplicates and the contact angles of three random spots on each duplicate were determined. 

### 3.4. Minimum Inhibitory Concentration (MIC) 

Four microbes, *Escherichia coli* (ATCC 8739, Gram-negative), *Staphylococcus aureus* (ATCC 6538, Gram-positive), *Pseudomonas aeruginosa* (ATCC 9027, Gram-negative), and *Candida albicans* (ATCC 10231, fungus) were used as representative microorganisms to challenge the antimicrobial functions of the antimicrobial compounds. Briefly, the bacteria were inoculated in Mueller Hinton (MH) broth (Oxoid Ltd., Basingstoke, UK) and fungi was grown in Yeast Mold (YM) broth (BD, Singapore). MICs were determined against bacteria with a concentration of 10^5^ CFU/mL and fungi with a concentration of 10^3^ CFU/mL using the broth micro dilution assay [23,32]. Media solutions containing microbial cells alone were used as control. The assay was performed in a 96-well plate. The lowest concentration of the antimicrobial compound at which no visible microbial growth, after 24 h of shaking incubation, was determined as MIC with visual observation. 

### 3.5. Japanese Industrial Standard (JIS Z 2801/ISO 22196) Method 

The JIS Z 2801/ISO 22196 method was used to determine the surface antimicrobial activity and bactericidal efficacy of the PS samples [33]. Before the test, *E. coli* and *S. aureus* were inoculated in 1/500 diluted Tryptic Soy (TS) broth (Oxoid Ltd., Basingstoke, UK), while *C. albicans* were inoculated in 1/500 diluted Yeast Mold (YM) broth (BD, Singapore). The cell suspensions were prepared in desired concentration at OD_600_ = 0.07 using a microplate reader (TECAN, Zürich, Switzerland), which corresponds to approximately 10^7^ CFU/mL. The solutions were then further diluted to obtain an initial inoculum concentration of 10^6^ CFU/mL and were placed onto the 2.5 cm × 2.5 cm PS sample surfaces and covered with a plastic film. After 24 h incubation at 37 °C, microbes on the test surfaces were washed off using 10 mL diluted media solution, and subsequently diluted and plated on LB agar plates. The resulting colonies were counted and the colony forming unit (CFU)/mL was determined.

### 3.6. Time-Kill Kinetic Assay 

A time-kill kinetic assay was performed to determine the antimicrobial efficiency of PS samples against *E. coli* according to JIS Z 2801/ISO 22196 test method with varying incubation periods, ranging from 0 h, 6 h, 8 h, 16 h, and 24 h. To summarize, bacterial cells were cultured in 1/500 diluted TSB and prepared into desired concentration in 1/500 diluted TSB. A 100 µL of inoculum with concentration of 10^6^ CFU/mL was seeded onto each 2.5 cm × 2.5 cm PS sample surfaces and incubated at 37 °C with varying incubation period. After incubation, the test surfaces were washed with diluted TSB and subsequently diluted before plated on LB agar plates. The colony forming unit (CFU)/mL was determined. All experiments were performed in triplicate, and the results were presented as mean and standard deviations.

### 3.7. GB 15979-2002 and Leaching Assay 

The leaching properties of PS*-1 were determined using a leaching assay and the results were compared with the GB 15979-2002 method. For the leaching assay, 0.1 g PS pieces were first placed in a 2 mL microcentrifuge tube containing 1 mL of PBS solution and incubated at 300 rpm for 1 h. After 1 h of incubation, the PS pieces were removed, and the *E. coli* suspension was added to the solution for a final concentration of 10^4^ CFU/mL. The solution that contained bacteria was incubated in a shaking incubator for another 1 h at 300 rpm. A 10-fold dilution was performed before plating 100 µL on an LB agar plate. The antibacterial efficiency of potential leached compounds and bacterial survivability were determined by counting the viable colonies on plate after 24 h. 

In contrast, for GB 15979-2002 test, 0.1 g of the PS pieces and the *E. coli* suspension were added together into a 2 mL microcentrifuge tube containing 1 mL of PBS solution and incubated at 300 rpm for 1 h. After 1 h incubation, dilution was performed, and the solution was plated on an LB agar plate for colony counting.

### 3.8. Scanning Electron Microscopy (SEM) Imaging of Bacteria Morphology Changes 

Bacteria *E. coli* suspension was prepared into a desired concentration 10^6^ CFU/mL in 1/500 diluted TSB. A volume of 25 µL bacterial suspension was added onto each of the 0.5 cm × 0.5 cm PS surfaces and incubated at 37 °C with an incubation time ranging from 0 h, 6 h, to 24 h. After incubation, the samples were lightly washed with PBS, followed by their fixation with 2.5% glutaraldehyde in PBS for 2 h at room temperature. Each sample was then soaked in a series of graded ethanol solution (25%, 50%, 75%, 95%, and 100%, 15 min each) for the dehydration of fixed bacteria. The treated samples were dried for 2 days at room temperature before being coated with a thin layer of platinum (≈5 nm) using high resolution sputter coater (JEOL, JFC-1600 Auto Fine Coating) for SEM imaging. The morphologies of bacteria were observed using field emission SEM JEOL JSM-7400F (Akishima, Tokyo, Japan).

### 3.9. Surface Structure Analysis with SEM and Atomic Force Microscopy (AFM) 

For a PS surface structure analysis using SEM, the samples were coated with thin platinum film before their observation via a field emission SEM JEOL JSM-7400F (Akishima, Tokyo, Japan).

For surface roughness determination, the topographic images of the PS surfaces were obtained using Nanoscope 9.7 Dimension ICON® Atomic Force Microscope (AFM) (Bruker, Billerica, MA, USA). Briefly, the PS samples were cleaned with deionised water and 70% ethanol before drying at 60 °C. The dried PS samples were subjected to a jet of compressed air to remove any particles on the surfaces. AFM tapping mode (phase contrast) measurements were performed with a scanning rate of 0.599 Hz, with an amplitude set point of 250 mV using a PointProbe-Plus^®^ Silicon-SPM-Sensor (PPP-NCHR-W) probe (k = 10–130 N/m, f = 204–497 kHz). For each sample, multiple images for 50 µm × 50 µm and 10 µm × 10 µm were acquired at 3 different locations per disk. NanoScope Analysis 2.0 software were used to analyse the AFM images and obtain the Mean Roughness (R_a_) and Root Mean Square Roughness (R_q_). R_a_ and R_q_ were calculated and averaged from more than 3 identical scan areas. 

### 3.10. Accelerated Aging and Shelf Life Test 

The antimicrobial efficiency and shelf life of PS*-1 was evaluated according to accelerated aging and a shelf life test according to the ASTM F1980-16 test method [34]. All PS samples were stored in a closed container with a vial of water to maintain a constant humidity of >95% and was placed in a 60 °C oven. The antimicrobial activity was evaluated according to the JIS Z 2801/ISO 22196 test method before aging (0 month), after 1 month, and after 2 months of accelerated aging. 

### 3.11. Cell Viability Assay 

A cell cytotoxicity assessment was conducted using an L929 mouse fibroblast cell viability assay. The L929 mouse fibroblast cells were cultured in DMEM complete media (supplemented with 10% (*v*/*v*) heat-inactivated fetal bovine serum (FBS), 100 U/mL penicillin and 100 µg/mL streptomycin) at 37 °C in 100% humidity and 5% CO_2_. PS controls and PS*-1 samples were prepared at 5 mg and 10 mg respectively and were incubated with the culture of mouse fibroblast cells in 96 well plates at 1.0 × 10^4^ cells/well for 24 h. The cell viability was determined using Alamar Blue reagent according to manufacturer’s protocol (Thermofisher Inc., Waltham, MA, USA)

### 3.12. Hemolysis Assay 

A hemo-compatibility evaluation was carried out using a red blood cell (RBC) hemolysis assay. The PS samples were prepared at 5 mg and 10 mg, respectively, and were incubated in 100 µL of diluted RBC in 96 well plate at 35 °C for 1 h. The plate was centrifuged at 2200 rpm/5 min and an aliquot of the supernatant was transferred to a new 96 well plate. The haemolytic activity was determined as a function of haemoglobin released by measuring OD_576_ at 100 µL of the supernatant. A control situation that contained only PBS (blank) was used as a reference for 0% hemolysis; TritonX100 treated samples were used as a positive control for 100% hemolysis. The formulation for the percentage hemolysis calculation is as follow:

(1) $$\mathrm{Hemolysis}\;\left(\%\right)=\;\frac{\mathrm{OD}576\;\mathrm{sample}-\mathrm{OD}576\mathrm{blank}}{\mathrm{OD}576\mathrm{TritonX}100-\mathrm{OD}576\mathrm{blank}}\;\times 100$$

## 4. Conclusions

A method of fabricating the inherent antimicrobial polystyrene, by decorating polymer chains with polyionic antimicrobial components, thereby leading to surface phase separation, was developed. The functionalized PS* exhibits a very rough surface with submicron scale structures of active components and presents enhanced hydrophilicity, demonstrating excellent antimicrobial efficiency. The interaction of bacteria cells with semi-free polyionic components on PS* surfaces via weak forces resulted in the high antimicrobial performance of PS*. Considering the relatively long shelf life and biocompatibility, the PS* can potentially be used for a wide range of applications, including in healthcare, medicine, and food packaging as green and sustainable self-disinfection materials.

## Figures and Tables

**Table 1:** Antimicrobial activities of PS controls, PS-PIM 45, and PS*-1 film surfaces (JIS Z 2801/ISO 22196 method).

Test Microbes	*E. coli*	*S. aureus*	*C. albicans*
0 h ^1^	6.43	6.05	5.50
24 h ^1^			
PS-comm	7.35	7.63	5.75
PS-control (T) ^2^	7.16	7.37	5.83
PS-PIM 45 (T)	6.90	7.62	5.72
PS*-1 (T)	0.00	0.00	5.64 (0.00) ^3^
0 h ^1^	6.68	6.26	5.70
24 h ^1^			
PS-comm	7.45	7.75	5.84
PS-control (C) ^2^	7.28	7.76	5.87
PS-PIM 45 (C)	7.66	7.50	5.97
PS*-1 (C)	0.00	0.00	6.10

^1^ Log_10_ (CFU/mL) of microbe. ^2^ (T) means sample prepared in toluene; (C) means sample prepared in chloroform. ^3^ No *C. albicans* growth was observed on PS*-3 (T) with 3 wt.% PIM-Vinyl after 24 h incubation.

## Data Availability

This present article reports only original data that were not published elsewhere.

## Supplementary Materials

The following are available online at https://www.mdpi.com/article/10.3390/ijms222212315/s1.
