# Supplementary material for: Soft Surface Nanostructure with Semi-Free Polyionic Components for Sustainable Antimicrobial Plastic
Source: Int J Mol Sci. 2021 Nov 15;22(22):12315. doi: 10.3390/ijms222212315 (PMC8621035; doi:10.3390/ijms222212315)
Supplement: Supplementary file 1 [file ijms-22-12315-s001.zip › ijms-1449197-supplementary.pdf]

Supporting Information

Soft surface nanostructure with semi-free polyionic components for sustainable antimicrobial plastic

Shook Pui Chan, Diane S. W. Lim, Arunmozhiarasi Armugam, Guangshun Yi, Yugen Zhang*

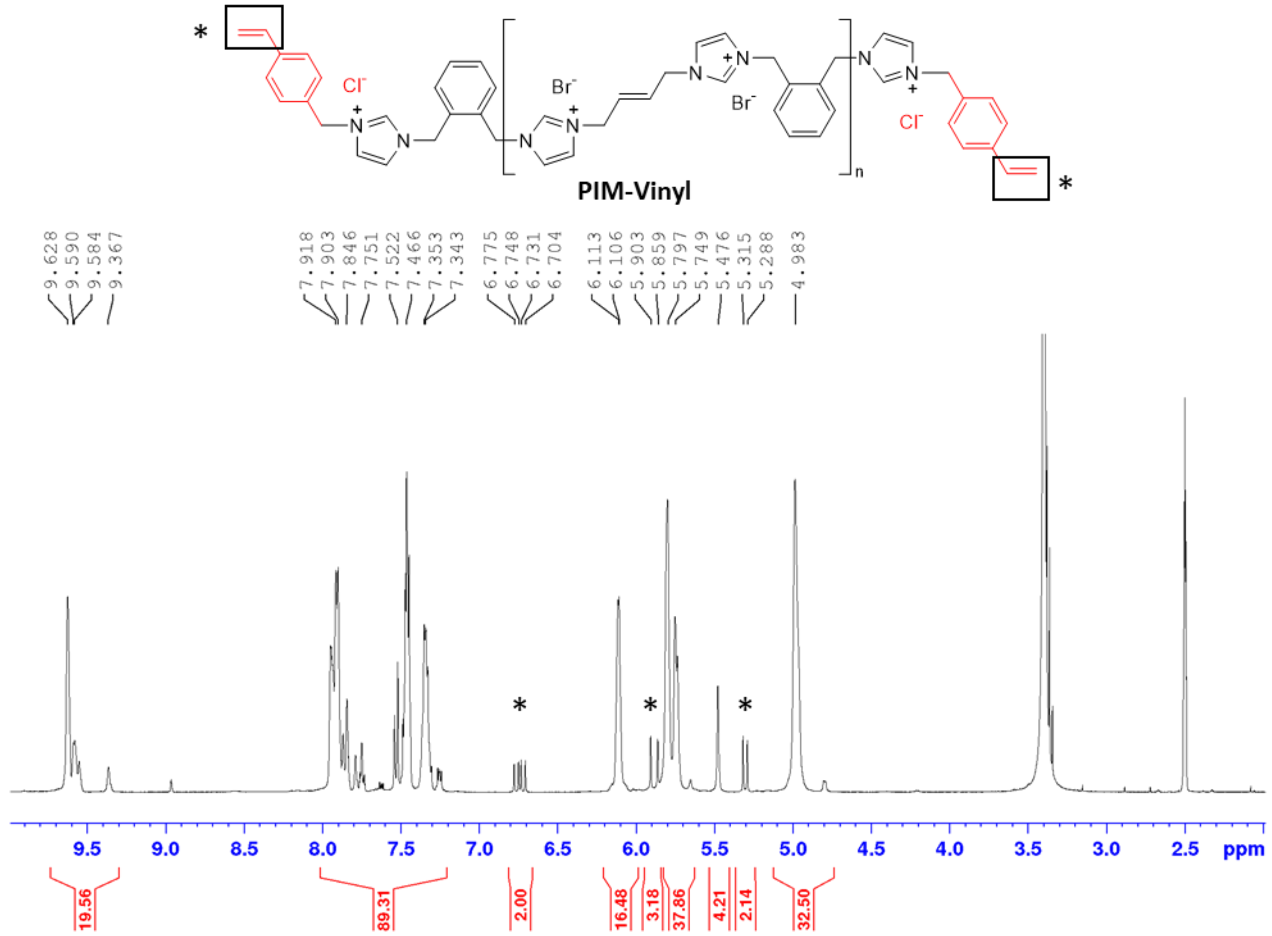

Figure S1. ^{1}H NMR (400 MHz, DMSO-d_6) spectrum and structure of PIM-Vinyl. The additional signals of vinyl terminal groups on PIM-45 were observed at ~6.74 ppm (dd), ~5.87 ppm (d), and ~ 5.32 ppm (d) respectively indicated as (*). dd, doublet of doublets; d, doublet.

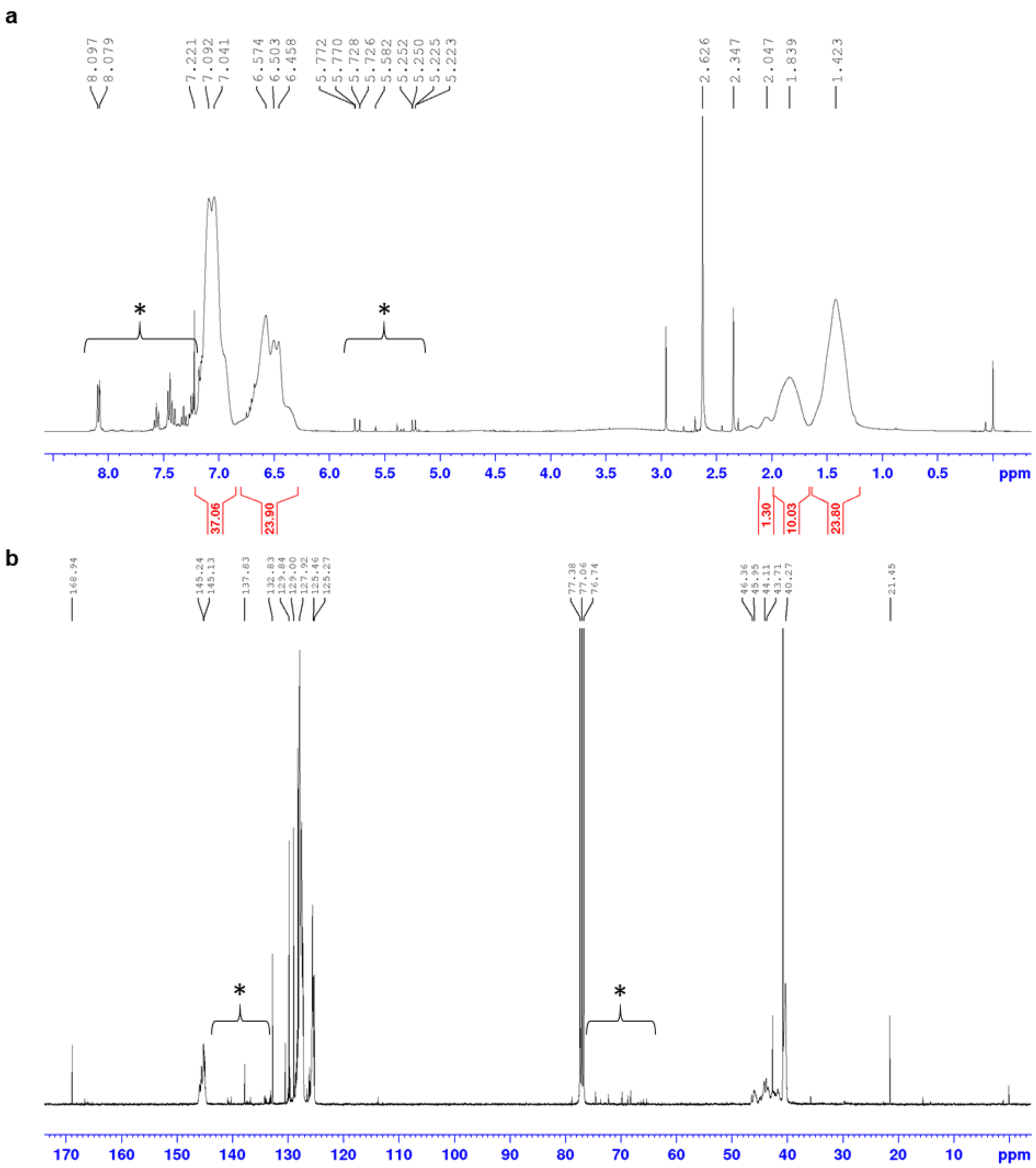

Figure S2. NMR spectrum of PS*-1. (a) ^{1}H NMR (400 MHz, $CDCl_3$) spectrum of PS*-1. Small peaks observed around 5.18 – 5.78 ppm; (b) ^{13}C NMR (100 MHz, $CDCl_3$) spectrum of PS*-1. Small peaks observed around 141.6 – 133.9 ppm and 65.3 – 79.0 ppm. (*) are assigned to the imidazolium rings and multiple benzylic CH_2 of PIM-Vinyl chain.

Table S1. Average molecular weight of synthesized polystyrene.

Sample	Mn	Mw	Polydispersity
PS-control	57224	127802	2.23
PS-PIM 45	57236	131452	2.29
PS*-1	49884	113705	2.28

Mn = Number average molecular weight; **Mw** = Weight average molecular weight; **Polydispersity index** = Mw/Mn

Table S2. Average contact angle of PS films.

Sample	Contact Angle (°)						
	Sample 1			Sample 2			Ave
PS-comm	97.8	98.3	100.1	96.5	97.0	102.5	98.7
PS-control (Toluene)	97.0	96.7	97.7	97.2	96.5	97.9	97.1
PS-PIM 45 (Toluene)	98.7	95.2	96.0	95.8	102.3	93.7	96.9
PS*-1 (Toluene)	87.1	91.8	86.1	88.7	90.4	90.1	89.0
PS-control (Chloroform)	97.9	95.4	94.4	94.9	97.7	93.8	95.7
PS-PIM 45 (Chloroform)	91.3	95.9	95.75	92.7	96	92.75	94.1
PS*-1 (Chloroform)	86.6	82.8	81.2	86.2	84.1	86.4	84.5

Table S3. Roughness average of surface, R_a of polystyrene samples obtained from AFM.

Sample	Average roughness, R_a (nm)	S.D.
PS-control (C)	6.733	1.802
PS*-1 (C)	55.175	19.308
PS-control (T)	3.805	0.191
PS*-1 (T)	102.900	19.940
PS#-1	64.950	0.354
PS-BZK C12	6.740	5.471

Table S4. Properties of PS* with different PIM-Vinyl loadings. Scale bars of AFM images, 10 μm.

PS*-X	AFM topographic images	Image R_q (nm)	Image R_a (nm)	JIS – *E. coli* Log reduction	JIS – *C. albicans* Log reduction
**X = 0.2 (0.2 wt. % of PIM-Vinyl)**	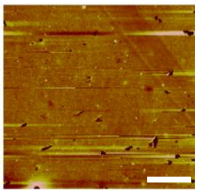	25.2	21.1	2	NA
**X = 0.5 (0.5 wt. % of PIM-Vinyl)**	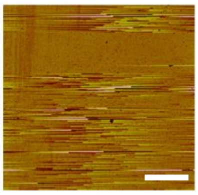	31.9	21.6	2	NA
**X = 1 (1 wt. % of PIM-Vinyl)**	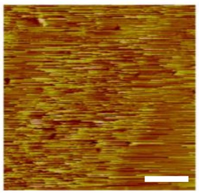	143.0	102.9	7 (Complete killing)	< 1
**X = 3 (3 wt. % of PIM-Vinyl)**	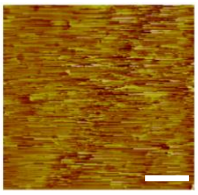	117.6	78.7	7 (Complete killing)	5 (Complete killing)

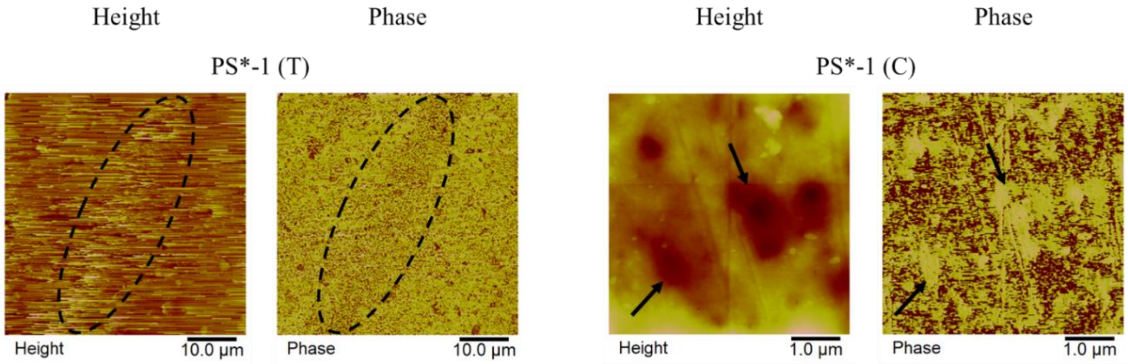

Figure S3. Height and phase images of PS*-1 in toluene and chloroform. The light and dark regions in height images of PS*-1 are in contrast with their respective phase images (as indicated in circle and arrows), reflecting that the nano-features are due to phase behaviour. The lighter region in both height images appeared to be darker in phase images which demonstrate the presence of hydrophilic components at areas with higher nanostructures.

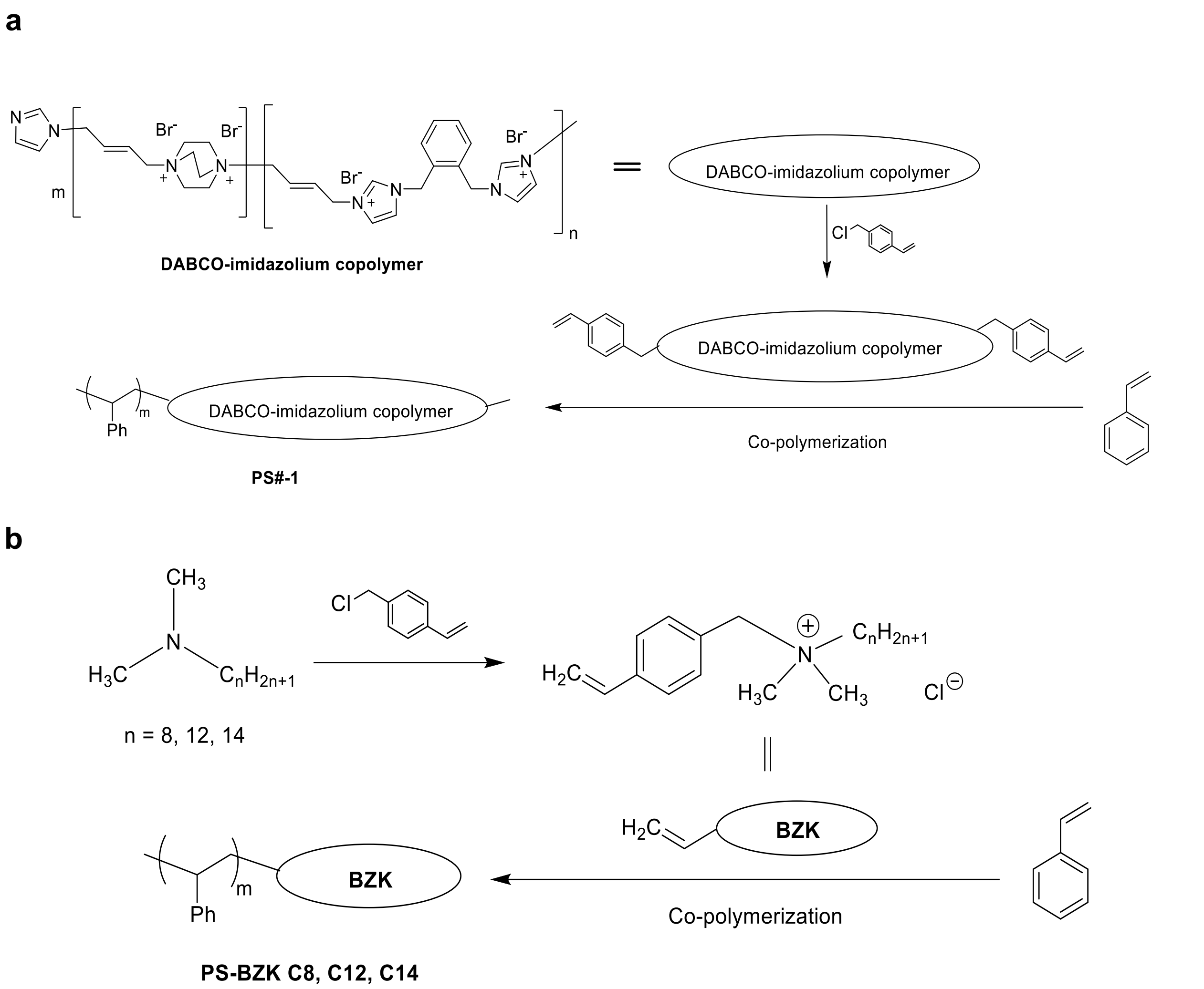

Scheme S1. PS incorporated with other antimicrobial compounds. (a) Modification of main-chain DABCO-imidazolium copolymer with a vinylbenzyl terminal group and subsequent polymerization with styrene to yield functionalized polystyrene (PS#-1); (b) Synthesis of quaternary ammonium compound benzalkonium chloride (BZK) with styrenyl moiety at one end and polymerization with styrene to yield functionalized polystyrene (PS-BZK C8, C12, and C14).

Table S5. Average minimum inhibition concentrations (MICs) of polyimidazolium compound PIM-45 and functionalized polyimidazolium compound PIM-Vinyl.

**Compound**	**MIC (μg/ml)**		
	E. coli	*S. aureus*	*C. albicans*
PIM-45	8	8	32
PIM-Vinyl	4	2	32

Table S6. Average minimum inhibition concentrations (MICs) of functionalized main-chain DABCO-imidazolium copolymer and benzalkonium chloride compounds.

Compound	MIC (μg/ml)		
	E. coli	*S. aureus*	*C. albicans*
DABCO-imidazolium copolymer	8	4	4
BZK C8	63	31	250
BZK C12	8	2	8
BZK C14	8	2	4

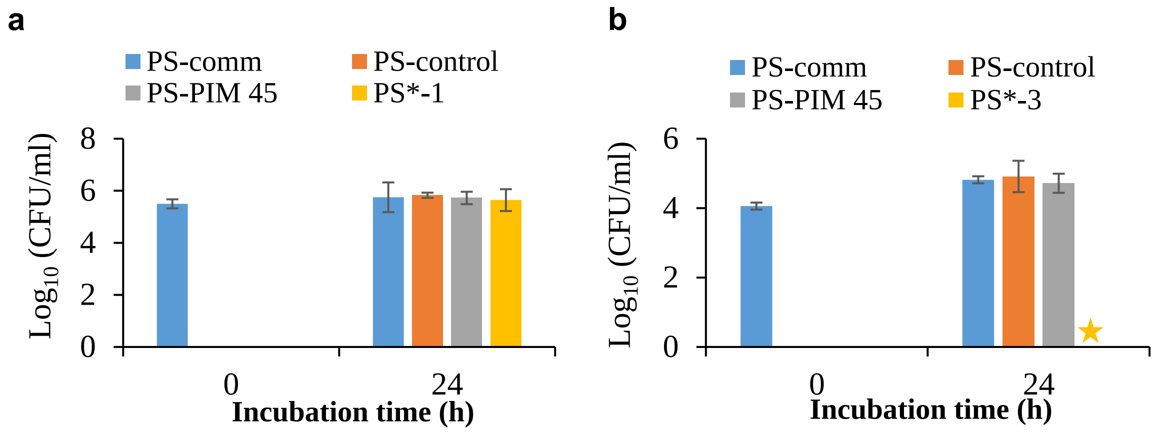

Figure S4. Antimicrobial activities of PS*-1 and PS*-3 against *C. albicans* (JIS Z 2801/ISO 22196 test method). (a) 1 wt. % PIM-Vinyl; (b) 3 wt. % PIM-Vinyl. PS*-3 with higher loading of antimicrobial compound PIM-Vinyl demonstrated good antifungal activity against *C. albicans*. All data are expressed as mean and standard deviations of triplicates. The standard deviation is shown by error bars. (★) indicates no colony observed.

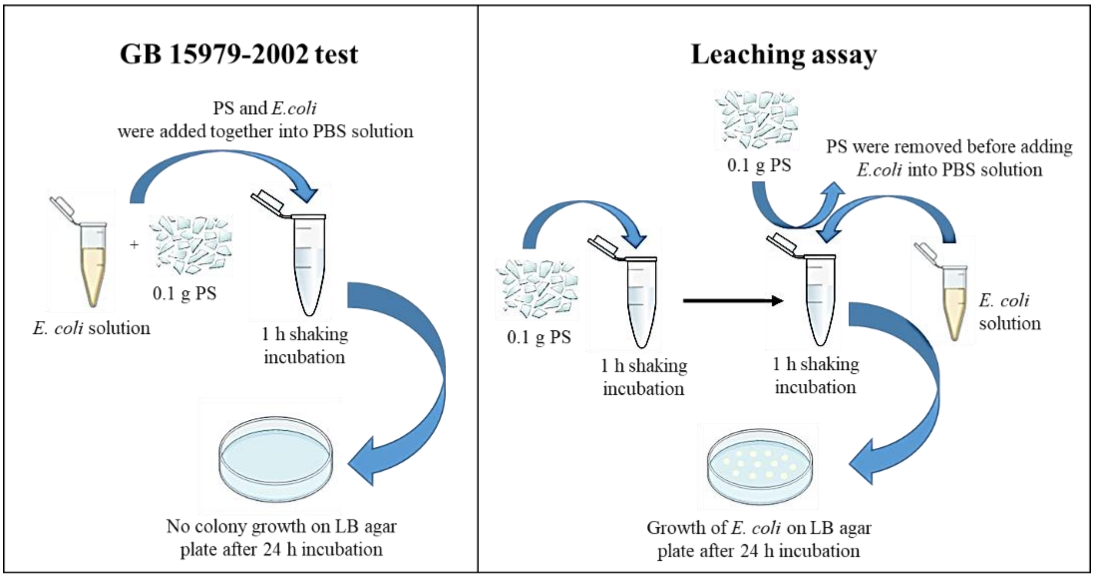

Figure S5. Illustration of leaching assay and modified GB 15979-2002 test of PS.

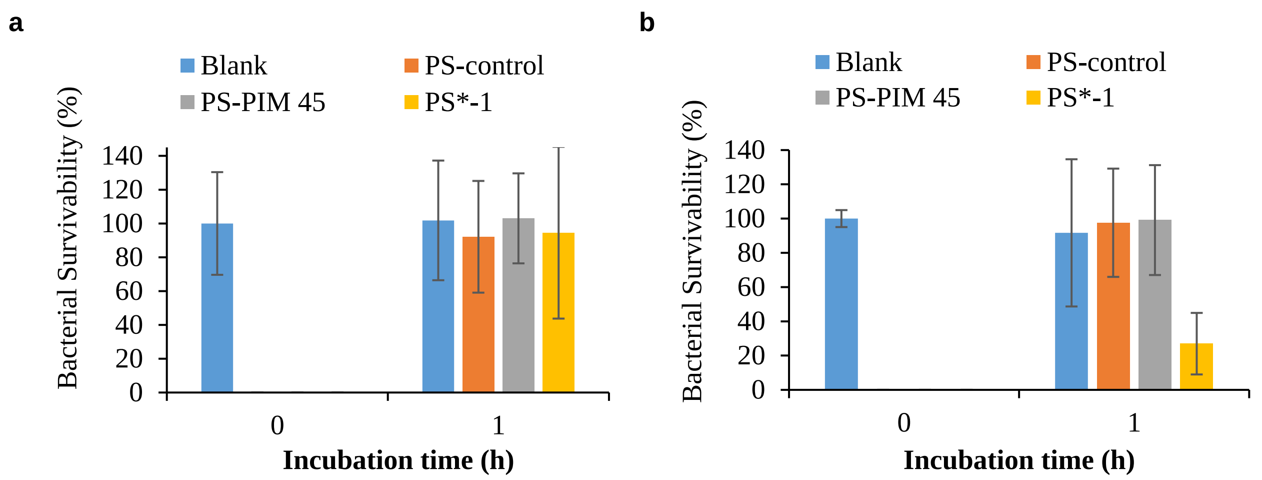

Figure S6. Leaching properties and antimicrobial activity of PS controls and PS*-1. (a) Leaching assay; (b) Modified GB 15979-2002 test method. No leaching of antimicrobial compound from PS*-1 was determined as high percentage bacterial survivability was observed from leaching test method. Low percentage bacterial survivability observed from GB test further confirmed the bacteria inhibition property of PS*-1 was due to surface contact killing. All data are expressed as mean and standard deviations of triplicates. The standard deviation is shown by error bars.

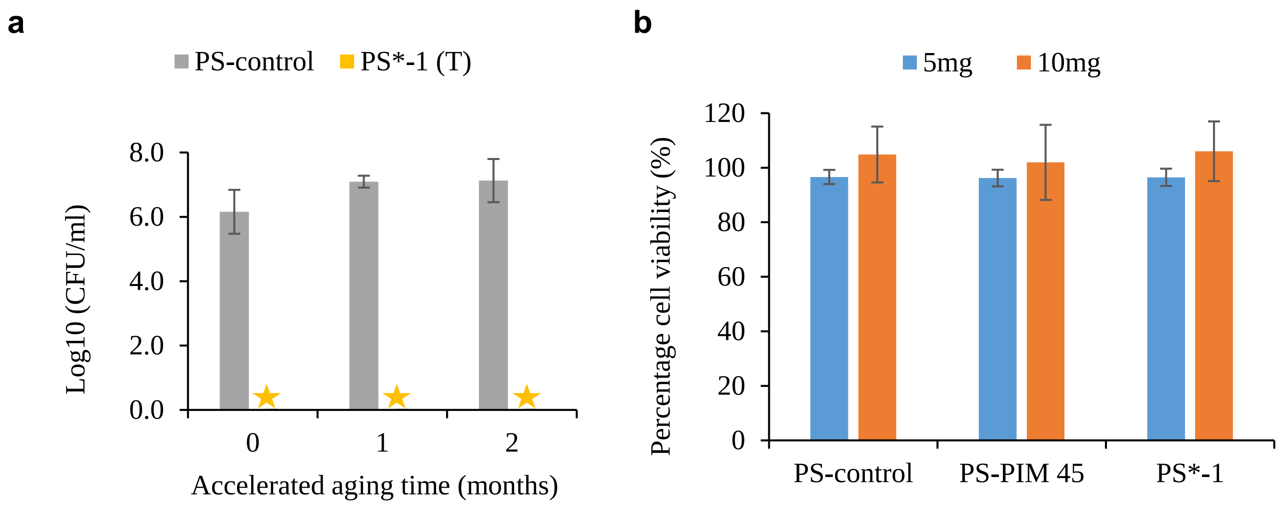

Figure S7. PS material properties studies. (a) Antimicrobial activities of PS*-1 against *E.coli* after accelerated aging for 2 months. PS*-1 exhibited excellent antimicrobial activity even after 2 months of aging; (b) Cyto-compatibility evaluation of PS-control, PS-PIM 45, and PS*-1 using mouse fibroblast L929 cells. PS*-1 did not show cytotoxicity effect on mouse fibroblast cells with percentage cell viability close to 100 %. All data are expressed as mean and standard deviations of triplicates. The standard deviation is shown by error bars. (★) indicates no colony observed.

Table S7. Hemo-compatibility assessment of PS films at 5 mg and 10 mg using red blood cells.

Sample	**5 mg**		**10 mg**	
	Average % hemolysis	SD	Average % hemolysis	SD
**PS-control**	-1.4531	0.4435	-0.8811	0.6013
**PS-PIM 45**	-1.2098	0.4459	-0.7233	0.7948
**PS*-1**	-1.4728	0.7087	-1.1901	1.2623
